# Long-Term Outcomes of the Aorfix™ Stent Graft in Japanese Patients with Severely Angulated Aortic Necks: A Single-Center Retrospective Study

**DOI:** 10.3390/jcm14248617

**Published:** 2025-12-05

**Authors:** Riha Shimizu, Makoto Sumi, Yuri Murakami, Masayuki Hara, Takao Ohki

**Affiliations:** 1 Department of Vascular Surgery, Saitama Prefectural Cardiovascular and Respiratory Center, 1696, Itai, Kumagaya 360-0197, Saitama, Japan; sumivascular@gmail.com (M.S.); yummmm66@gmail.com (Y.M.); haharappy@hotmail.com (M.H.); 2 Department of Cardiac and Vascular Surgery, Dokkyo Medical University Nikko Medical Center, 145-1, Moritomo, Nikko 321-1298, Tochigi, Japan; 3 Division of Vascular Surgery, Department of Surgery, The Jikei University School of Medicine, 3-19-18, Nishishinbashi, Minato-ku, Tokyo 105-8471, Japan; takohki@msn.com

**Keywords:** abdominal aortic aneurysm, endovascular aortic aneurysm repair, long-term clinical outcome, angulated aortic neck, stent graft

## Abstract

**Background/Objective**: Endovascular aneurysm repair (EVAR) using Aorfix (Lombard Medical, Inc., Irvine, CA, USA)^TM^ has shown excellent outcomes, even in cases of abdominal aortic aneurysm with highly angulated aortic necks (≥60°). However, long-term outcomes for Japanese patients remain unknown. In this study, we aimed to investigate the performance of Aorfix^TM^ in Japanese patients with highly angulated aortic necks. **Methods**: Among 114 patients in whom Aorfix^TM^ was used for EVAR at a single institution from October 2014 to October 2021, 105 patients without rupture or infection were retrospectively reviewed. They were classified into the following two groups: those with proximal neck angulations of ≥60° and <60°. Endpoints included technical success, long-term survival, freedom from aneurysm-related mortality, and freedom from reintervention. **Results:** Among 105 cases reviewed, 54 and 51 had proximal neck angulations of <60° and ≥60°, respectively. The <60° and ≥60° groups had a mean neck angulation of 30.7° (median 30°, range 10–56°) and 80.3° (median 77°, range 60–110°), respectively. The ≥60° group had significantly increased operation time (*p* = 0.034), volume of contrast agent used during the operation (*p* = 0.0301), and duration of fluoroscopy during the operation (*p* < 0.0001); however, the rates of additional renal artery stenting, cuff placement, and access site complications did not differ between the groups. There were also no differences in the incidence of aneurysm enlargements, secondary intervention, and endoleaks incidence. **Conclusions**: EVAR with Aorfix^TM^ achieved satisfactory results in Japanese patients with severe and mild/moderate proximal neck angulation.

## 1. Introduction

Endovascular aneurysm repair (EVAR) is less invasive than open surgery and provides superior early outcomes in the treatment of abdominal aortic aneurysm (AAA) [1,2,3]. However, EVAR is limited by anatomical constraints that can increase the risk of aneurysm-related complications [1,2,3]. One of these anatomical restrictions is proximal neck angulation, which is associated with Type 1a and Type 3 endoleaks, as well as aneurysm enlargement, ruptures, and/or open conversions [4,5,6]. Therefore, the instructions for use (IFUs) of devices commercialized by other manufacturers in Japan recommend EVAR only for patients with proximal neck angulation <60°.

The Aorfix^TM^ bifurcated aortic stent graft (Lombard Medical, Inc., Irvine, CA, USA), designed to overcome this anatomical restriction, was the fifth abdominal stent graft to receive regulatory approval for use in Japan in August 2014. This nitinol/polyester stent graft has a ring-shaped body and helical-limb structure, and its terminal end resembles a fish-mouth shape, allowing transrenal placement of the stent using the vessel above the renal artery as the fixation site.

Aorfix™ has been approved for use in cases with proximal neck angulation of ≤90° [7,8], in accordance with the Aorfix Bifurcated Safety and Performance Trial: phase II, angulated vessels (ARBITER-II) trial conducted in the United Kingdom and the Prospective Aneurysm Trial: High Angle Aorfix Bifurcated Stent Graft (PYTHAGORAS) trial conducted in the United States, which reported favorable 5-year outcomes [9]. However, data regarding the use of Aorfix^TM^ and its long-term outcomes in the Japanese population remain limited. We aimed to compare the short- and mid-to-long-term outcomes of Aorfix^TM^ in Japanese patients with proximal neck angulations of ≥60° versus <60°, treated at a single institution.

## 2. Materials and Methods

### 2.1. Patients

This retrospective study included patients with AAA in whom Aorfix^TM^ was used for EVAR procedures at our hospital from October 2014 to October 2021.

EVAR was performed in patients at higher risk for open surgery, including elderly people and those with comorbidities. The inclusion criteria for using EVAR were AAA ≥50 mm or iliac artery aneurysm (IAA) ≥30 mm. In contrast, cases with rupture (including those with impending rupture), those with infected or inflammatory aneurysms, and those with isolated IAA were excluded from the study. Our study was approved by the Institutional Review Board of our institution (No. 2022-013).

The IFU for Aorfix^TM^ confirms that it can be used to treat AAAs with the following characteristics: proximal neck length >15 mm, proximal neck diameter between 19 and 29 mm, distal landing zone diameter 9–19 mm, and distal landing zone length >15 mm.

All patients received general anesthesia, and surgical access was via the femoral arteries.

### 2.2. Follow-Up Protocol

Postoperatively, contrast-enhanced computed tomography (CT) and ultrasonography were performed to assess the arterial and venous phases at 1, 6, and 12 months. Non-contrast CT and ultrasonography were used in patients with poor renal function and in those who were allergic to the contrast agent. For hydration, some patients were hospitalized for 2–3 days, as required, prior to contrast CT. The subsequent follow-up course for these patients was monitored annually using non-contrast CT and ultrasonography. Sac expansion was defined as a 5 mm increase in sac diameter, while sac shrinkage was defined as a decrease in sac diameter of 5 mm. In patients without sac enlargement, low-flow endoleaks detected only by ultrasonography were managed conservatively. Reintervention was indicated for type 1 or 3 endoleaks, or for type 2 endoleaks associated with aneurysm sac expansion ≥ 5 mm/year. In addition, survival and mortality states were obtained through retrospective review of our institutional electronic medical records. For patients who discontinued routine follow-up, survival was further confirmed using hospital visit records within our medical network and, when necessary, by direct contact with the patients or their families.

### 2.3. Outcomes

The aim of the study was to compare rates of technical success, presence or absence of short- and long-term (5 years) endoleaks, change in sac size, rates of reintervention, and mortality after EVAR with Aorfix^TM^ between patients with highly angulated aortic necks (≥60°) and patients with standard aortic necks (<60°).

### 2.4. Statistical Analysis

Continuous variables were expressed as mean ± standard deviation or as median with range, as appropriate, and compared between the two groups using Student’s *t*-test or the Mann–Whitney U test. Categorical variables were expressed as numbers and percentages and compared using the χ^2^ test or Fisher’s exact test, as appropriate.

Freedom from all-cause mortality and freedom from reintervention were analyzed using the Kaplan–Meier method, and differences between ≥60° and <60° groups were assessed using the log-rank test. A *p*-value of <0.05 was considered to indicate a statistically significant difference. Missing or incomplete follow-up data were handled using an available case approach. Patients with partial follow-up were included in the analysis for the period in which reliable data were available, and were treated as censored observations in survival analyses. All statistical analyses were performed using JMP version 14.0 (SAS Institute, Cary, NC, USA).

## 3. Results

### 3.1. Overview

Of the 114 cases that underwent EVAR using Aorfix^TM^ from October 2014 to October 2021, we excluded cases with infection or rupture, and the remaining 105 cases were included in the analysis.

The mean observation period was 34 months (median 34, range 1–83), and the cases followed were 90 at 1 year, 69 at 2 years, 54 at 3 years, 30 at 4 years, and 16 cases at 5 years. Some patients were not followed regularly due to restricted hospital visits during the COVID-19 pandemic, and several patients had not yet reached the scheduled follow-up period at the time of data collection.

Of the 105 participants, 90 were males and 15 were females, with a mean age of 75.5 (median 77, range 50–95) years. Patients’ characteristics are shown in Table 1. The <60° group included 54 cases, and the ≥60° group included 51 cases. Patients in the ≥60° group had a lower body mass index (*p* = 0.0029) and were significantly older (*p* = 0.0497); moreover, the proportion of female patients was higher in this group (*p* = 0.0354). No differences in the use of antiplatelet or anticoagulant drugs were observed between the two groups (*p* = 0.4271, *p* = 0.1496).

Baseline characteristics of aneurysms are shown in Table 2. Maximum AAA sac diameter was 51.6 ± 7.1 mm (median 50 mm, range 44–75 mm), the proximal neck diameter was 20.2 ± 2.7 mm (median 20 mm, range 15–27 mm), and the proximal neck length was 34.5 ± 15.1 mm (range 8–86 mm). Preoperative maximum sac diameter did not differ between the groups (<60°: 50.3 ± 5.6 mm vs. ≥60°: 53.0 ± 8.2 mm; *p* = 0.056). No significant differences were found between the groups in terms of neck diameter (<60°: 20.7 ± 2.5 mm vs. ≥60°: 19.6 ± 2.9 mm; *p* = 0.0521) or neck length (<60°: 33.3 ± 13.9 mm vs. ≥60°: 35.9 ± 16.4 mm; *p* = 0.3771).

The mean neck angulation of all cases was 54.8° ± 29.2°, with 30.7° ± 15.1° in the <60° group (54 cases) and 80.3° ± 15.6° in the ≥60° group (51 cases). In addition, 15 cases (29.4%) in the ≥60° group had a neck angulation of ≥90°.

### 3.2. Primary Technical Success and Early Complications

The technical outcomes are summarized in Table 3. The technical success rate was 100%. The operation time was significantly longer in the ≥60° group than in the <60° group (168.9 ± 50.9 min vs. 140.1 ± 47.4 min; *p* = 0.0034). The contrast agent volume was also significantly higher in the ≥60° group (189.5 ± 52.0 mL vs. 167.7 ± 47.2 mL; *p* = 0.0301). Furthermore, the fluoroscopy time was significantly longer in the ≥60° group (56.1 ± 23.0 min vs. 38.0 ± 17.2 min; *p* < 0.0001).

A proximal extension cuff was used in eight cases (7.6%), and no difference was found in the rate of proximal extension cuff use between the two groups (*p* = 0.1129). Renal artery stents were inserted in seven cases (6.7%) where the renal artery was covered; however, no significant differences were found between the two groups (*p* = 0.2650).

Access-related complications were noted in 17 cases (16.2%), but no significant differences due to proximal neck angulation were observed between the two groups (*p* = 0.1440). Since the internal iliac artery (IIA) was covered in five cases (4.8%) (<60° group: three cases; ≥60° group: two cases) by the stent graft, the bare-metal stent was placed in the external iliac artery (EIA). Access route dissection was observed in two cases (3.9%) in the ≥60° group, and EIA stenosis was observed in ten cases (9.5%) (<60° group: three cases; ≥60° group: seven cases).

Early complications are presented in Table 4. Aortic intramural hematoma was observed in a postoperative CT scan within 30 days in one case (2.0%), with a proximal neck angulation of ≥60°, which improved after complete rest and antihypertensive therapy.

Type 1a endoleak was observed in one case in the ≥60° group, and Type 2 endoleak was observed in 25 cases (23.8%); however, no significant differences were observed between the two groups (<60° group: 12 (22%); ≥60° group: 13 (25.5%); *p* = 0.4367). There were no cases of migration, early deaths, or open conversion.

### 3.3. Late Complications and Endoleaks

The mean follow-up period after EVAR was 34.0 ± 20.1 months.

**Figure 1:**
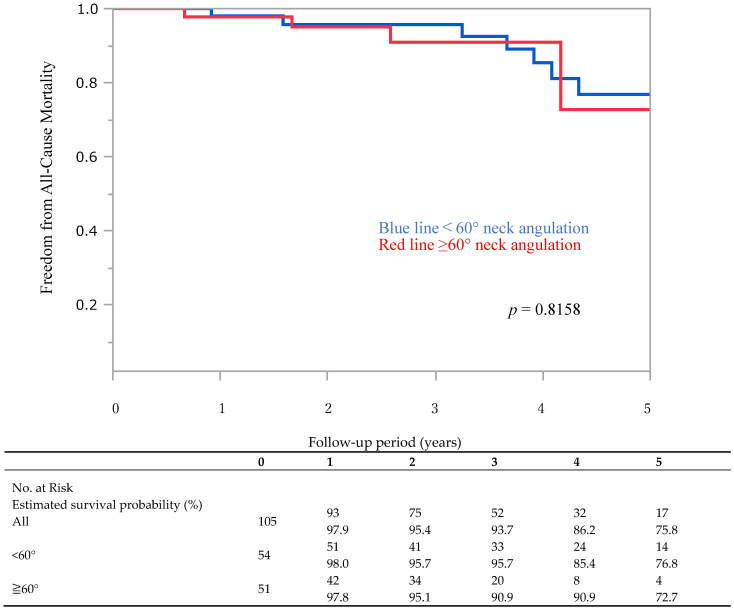
Kaplan–Meier survival curve stratified by neck angulation (<60° vs. ≥60°). There was no significant difference between the groups (*p* = 0.8158). The table below the figure shows the number of patients at risk and estimated cumulative survival probability at each yearly time point. In this figure, the red line represents patients with neck angulation ≥60°, and the blue line represents those with neck angulation <60°.

The 5-year overall survival rate was 75.8%. In the <60° group, the overall survival was 98.0% at 1 year, 95.7% at 3 years, and 76.8% at 5 years, whereas in the ≥60° group, the overall survival was 97.9% at 1 year, 90.9% at 3 years, and 72.7% at 5 years; there were no significant differences (*p* = 0.8158) between the two groups’ overall survival rates (Figure 1). The table below Figure 1 shows the number of patients at risk at each yearly time point. Additionally, no aneurysm ruptures were observed in any of the cases.

**Figure 2:**
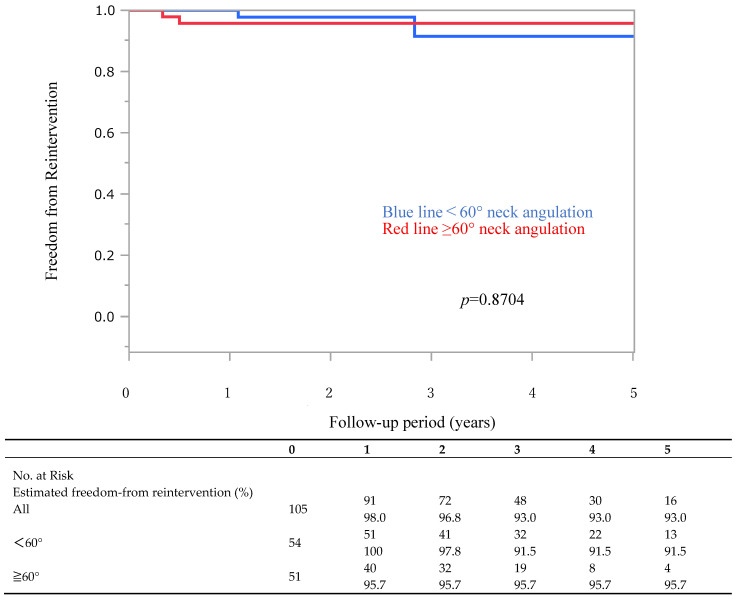
Kaplan–Meier curve showing freedom from reintervention stratified by neck angulation group (<60° vs. ≥60°). There was no significant difference between the groups (*p* = 0.8704). The table below indicates the number of patients at risk and estimated freedom from reintervention at each yearly time point. In this figure, the red line represents patients with neck angulation ≥60°, and the blue line represents those with neck angulation <60°.

Overall freedom from secondary treatment (Figure 2) was 98.0% at 1 year, 93.0% at 3 years, and 93.0% at 5 years. In the <60° group, freedom from secondary treatment was 100% of cases at 1 year, 91.5% at 3 years, and 91.5% at 5 years, whereas in the ≥60° group, it was 95.7% at 1 year, 95.7% at 3 years, and 95.7% at 5 years. There was no significant difference between the two groups (*p* = 0.8704). The table below Figure 2 indicates the number of patients at risk at each yearly point.

Complications other than endoleaks occurred in five cases during follow-up (4.9%). Three cases of EIA stenosis were treated with stent placement (two in the <60° group and one in the ≥60° group). Ultrasonography revealed elevated peak systolic velocity; however, all patients remained asymptomatic. Two cases with renal artery stenosis in the ≥60° group underwent treatment with a balloon-expandable stent.

Regarding reintervention for endoleak, two cases in the <60° group had Type 2 or Type 3b endoleaks, and one case had a Type 1b endoleak. Aneurysm enlargement due to Type 2 endoleaks was seen in two patients in the <60° group, and both underwent coil embolization of lumbar arteries. No reintervention was needed in the ≥60° group.

Changes in aneurysm size are presented in Table 5. In all cases, shrinkage of ≥5 mm occurred in 57.8% at 1 year, 551.1% at 2 years, 57.4% at 3 years, 53.3% at 4 years, and 68.7% at 5 years post-procedure. In the <60° group, shrinkage was observed in 54.0% at 1 year, 57.9% at 2 years, 62.5% at 3 years, 60.0% at 4 years, and 76.9% at 5 years, whereas aneurysm shrinkage was observed in 62.5% at 1 year, 51.6% at 2 years, 50.0% at 3 years, 40% at 4 years, and 33.3% at 5 years in the ≥60° group; no statistically significant differences were observed between the two groups.

Aneurysm enlargement of ≥5 mm was observed in 0% at 1 year, 5.6% at 3 years, and 6.3% at 5 years. In the <60° group, sac enlargement occurred in 2.6% at 1 year, 3.1% at 3 years, and 7.7% at 5 years, whereas in the ≥60° group it occurred in 0% at 1 year, 9.1% at 3 years, and 0% at 5 years.

Endoleak incidence is summarized in Table 6. Type 2 endoleaks were observed in 26.7%, 21.4%, 22.2%, 25.8%, and 18.8% of all patients at 1, 2, 3, 4, and 5 years, respectively. When stratified by neck angulation, the <60° group showed incidences of 26.5%, 23.7%, 21.9%, 23.8%, and 15.4%, whereas the ≥60° group showed incidences of 26.8%, 19.4%, 22.7%, 30.0%, and 33.3% at 1, 2, 3, 4, and 5 years, respectively. There were no significant differences between the two groups at any time point (1 year: *p* = 0.9745; 2 years: *p* = 0.6635; 3 years: *p* = 0.9410; 4 years: *p* = 0.7149; 5 years: *p* = 0.4972).

The proportions of patients receiving antiplatelet or anticoagulant therapy did not differ between the ≥60° and <60° groups (*p* = 0.5271, 0.1496). In addition, the occurrence endoleaks at 1 year was not significantly different according to medication use (*p* = 0.4610, 0.1612). However, aneurysm sac shrinkage was significantly greater in patients who were not receiving antiplatelet (*p* = 0.0302).

Approximately 38% of patients experienced an interruption or loss to follow-up, or had not yet reached the scheduled follow-up period at the time of data collection. This was mainly due to difficulty in attending regular hospital visits during the COVID-19 pandemic, which may have influenced the completeness of long-term data.

## 4. Discussion

A previous randomized controlled trial comparing EVAR with laparotomy found that EVAR had a lower mortality [1,2]; however, EVAR has been reported to increase the rate of aneurysm-related mortality and secondary interventions in the mid to long term [2,3,4,5,10]. In cases with severe neck angulation, an inadequate sealing zone between the stent graft and aorta may increase the incidence of endoleaks and aneurysm enlargement [4,9,11]. Among patients with AAA in the United States, it is estimated that up to 12% of men and 26% of women may have aortic neck angulation >60°, making the applicability of existing EVAR stent graft technology “off-label” for approximately 20% of all patients with AAA [8,12]. Similarly, among Japanese patients, a study reported that cases with severe neck angulation were more likely to experience sac enlargement [13].

The ARBITER-II and PYTHAGORAS trials observed the same rate of mortality and complications among normal and severe neck angulation cases [7,8]; they also demonstrated the safety and efficacy of Aorfix^TM^ for cases with severe neck angulation, leading to approval for use of the device for proximal neck angulation of ≤90°. A 5-year follow-up study demonstrated the effectiveness of the device in cases with severe neck angulation [9].

According to a study on cases with severe neck angulation where other commercially available devices (AneuRx (Medtronic Inc., Santa Rosa, CA, USA), Excluder (W. L. Gore & Associates, Flagstaff, AZ, USA), Zenith (Cook Medical Inc., Bloomington, IN, USA), and Talent (Medtronic Inc., Santa Rosa, CA, USA), were used, 22% of cases developed Type 1a endoleaks requiring treatment by cuff placement [14]. The rate of incidence of requirement of renal artery stent or cuff placement was also high (28.6%) in the EVAR 1 Trial [15]. The PYTHAGORAS trial on patients with neck angulation of ≥60° reported a rate of 18.2%, which was lower than the rate of Type 1a endoleaks seen with other devices [8]. As Aorfix^TM^ can contour the angulation, fewer cases may require cuff placement and renal artery stents. The present study revealed no significant differences in renal artery stents or cuff placement between the two groups with different neck angulation. In the ≥60° group, only 11% of cases required cuff placement; however, the operation time (*p* = 0.0034), contrast volume (*p* = 0.0301), and fluoroscopy time (<0.0001) were significantly increased. These findings may be attributed to the Aorfix^TM^ ring-shaped configuration and fish-mouth-shaped proximal end, which require precise C-arm orientation to avoid covering the renal and superior mesenteric arteries while securing fixation above the renal artery. As reported by Albertini et al., device positioning and angulation take longer in cases with neck angulation ≥60°, and renal artery stenting may be required even in cases without severe angulation [16]. In our study, five patients in the <60° group required renal artery stenting due to proximal migration of the stent graft, underscoring the importance of careful monitoring of proximal positioning during deployment.

Intraoperative access problems occurred in 16.2% of cases, with no differences in the incidence of iliac constriction and occlusion between the two groups with different neck angulation. Aorfix™ has an external diameter of 20 Fr, which is larger than that of similar devices by other manufacturers and has a stiffer delivery system; thus, access route dissection may be more common with Aorfix™ than other devices. Access route dissection occurred in two cases in the ≥60° group in the present study. However, 25% of the ultrasonography follow-ups were reported to have femoral artery or EIA dissection [17,18], which may not be caused by the device. Furthermore, previous studies have demonstrated that Asian patients undergoing EVAR tend to have greater iliac tortuosity and smaller external iliac artery diameters, both of which are associated with an increased risk of vascular access related complications [19]. Consistent with these findings, similarly small EIA diameters have been reported in Japanese EVAR patients [20]. Taken together, these anatomical characteristics indicate that particular caution is required when using the relatively large and stiff 20 Fr Aorfix^TM^ delivery system in Japanese and other Asian populations.

Aorfix™ may unintentionally cover IIA or cause insufficient distal expansion due to its fish-mouth shape [18]. Of the 48 cases, 8 (16.7%) were reported to have developed IIA occlusion [18]; moreover, IIA occlusion was observed in 2 cases in the ARBITER 2 trial, both of which had neck angulation of ≥90° [3]. In the PYTHAGORAS trial, two cases had long and narrow terminal aortas for which stents were placed to treat the compression of the ipsilateral limb at the level of the cannulation gate, three cases had poor dilation of the overlap region, and one case had occlusion that occurred due to an oversized limb. At our hospital, IIA occlusion occurred in five cases (4.8%) (<60° group: three cases; ≥60° group: two cases), and EIA stenosis occurred in ten cases (9.5%) (<60° group: three cases; ≥60° group: seven cases). Deployment requires considerable caution and a learning curve due to the challenge of determining graft length for winding the iliac artery [3]. In the present study, all cases showed an improvement after iliac stent placement, and no cases of postoperative iliac occlusion were observed. This is possibly because the Aorfix^TM^ is a stent graft with a ring shape and a non-bulky lumen when oversized [12]. Therefore, stent implantation could be a preventive option for intraoperative dissection, stenosis, or unexpected EIA landing.

A previous study that analyzed predictors of all-cause mortality after EVAR reported a 5-year mortality rate of 31–32% [21,22]. In the present study, the 5-year mortality rate was approximately 25%, which was slightly lower than that reported in their study. This difference may reflect variations in patient background, device selection, or follow-up management. Nevertheless, the overall trend is consistent with previous findings, suggesting that long-term survival after EVAR using the Aorfix^TM^ in our population was acceptable and comparable to previous reports. Regarding aneurysm enlargement, our findings were similar to those reported in the PYTHAGORAS trial: ≥5 mm in 1% at 1 year, 7% at 3 years, and 12% at 5 years [9]. In comparison, aneurysm enlargement of ≥5 mm occurred in 15% at 5 years with Excluder [23], whereas it occurred in 18% at 3.6 years with Zenith and Endurant [24]. The use of Zenith in Japanese cases is reportedly associated with aneurysm enlargement of ≥5 mm in 25% at 5 years and 33% at 10 years [25], which is less than that observed in cases using devices by other manufacturers. The predictive factors for aneurysm enlargement include preoperative sac diameter, presence of proximal neck angulation, and short neck [13]. Moreover, cases with proximal neck angulation of ≥60° in the PYTHAGORAS trial showed enlargement in 2% at 1 year, 7% at 3 years, and 15% at 5 years [9], indicating a higher tendency than that in cases with neck angulation of <60°; however, there were no significant differences between the two groups. At our hospital, expansion was also observed more frequently in cases with severe neck angulation, but no statistically significant difference was observed between groups. The Japanese data reported such enlargement in 2.6% of cases at 6 months, 4.4% of cases at 3 years, and 23.3% of cases at 5 years [13]. These findings suggest that Aorfix™ may be a favorable option for Japanese patients with severe neck angulation.

The PYTHAGORAS trial indicated that Types 1, 3, and 2 endoleaks were not significantly affected by proximal neck angulation [9], and the same may be reported for the current investigation. Using the existing devices (Ancure (Guidant Corp., Indianapolis, IN, USA), Powerlink (Endologix Inc., Irvine, CA, USA), AneuRx (Medtronic Inc., Santa Rosa, CA, USA), Excluder, Zenith, and Lifepath (Edwards Lifesciences LLC, Irvine, CA, USA)), it was confirmed that the endoleak blood flow increased in cases with proximal neck angulation of >30°. However, the blood flow with the Aorfix^TM^ was not affected by the neck angle, and endoleak was not increased [26]. Therefore, Aorfix^TM^ is considered to have better flexibility than Zenith for cases with neck angulation of ≥60° [27].

Additionally, Type 2 endoleak in Aorfix^TM^ has been reported for 13% at 1 year, 8% at 3 years, and 9% at 5 years [9]; however, the result of Type 2 endoleaks in our hospital was higher than the reported data. Possible explanations include: (1) the reported Type 2 detection rate in the Japanese population is 28.1% [28], (2) color Doppler may be more sensitive than CT in detecting endoleaks [29], and (3) large-scale Japanese EVAR data have shown type 2 endoleak rates of 16.2–16.6% at discharge, indicating that our findings remain within the range reported in Japanese cohorts [14].

The limitations of the current study include the fact that it was a single-center non-randomized study and case numbers with longer follow-up data were limited. In addition, the overall sample size was small, which may have reduced the statistical power and imposed limitations on drawing definitive comparative conclusion. We continue to follow up on these cases to increase the number available for a more comprehensive analysis in the future. In addition, because annual surveillance mainly relied on non-contrast CT and ultrasonography, low-flow endoleak may have been undetected [30,31]. Not all patients were followed up because some could not attend regular hospital visits during the COVID-19 pandemic, and several had not yet reached the corresponding follow-up by the end of data collection. This may have limited the robustness of long-term outcome assessments, including reintervention and endoleak rates.

## 5. Conclusions

Aorfix™ demonstrated to be a safe and effective device even in anatomically challenging cases with severe proximal neck angulation in a Japanese cohort, demonstrating comparable outcomes to standard neck anatomy.

## Figures and Tables

**Table 1:** Baseline characteristics of patients undergoing EVAR with Aorfix™, stratified by proximal neck angulation (<60° vs. ≥60°).

	Aorfix (N = 105)	<60° (n = 54)	≥60° (n = 51)	*p*-Value
Age, years	75.5 ± 8.1	73.9 ± 8.4	77.1 ± 7.6	0.0497
Female sex, no. (%)	15 (14.3)	4 (7.4)	11 (21.6)	0.0354
Body mass index, kg/m^2^	23.4 ± 3.8	24.2 ± 4.0	22.7 ± 3.6	0.0455
Obesity, no. (%)	32 (30.5)	20 (37.0)	12 (23.5)	0.0029
Coronary artery disease, no. (%)	36 (34.3)	21 (38.9)	15 (29.4)	0.3056
Cerebrovascular disease, no. (%)	28 (26.7)	14 (25.9)	14 (27.5)	0.8598
Hypertension, no. (%)	85 (81.0)	42 (77.8)	43 (84.3)	0.3924
Lower extremity artery disease, no. (%)	4 (3.8)	2 (3.7)	2 (3.9)	0.9535
Cancer, no. (%)	27 (25.7)	12 (22.2)	15 (29.4)	0.3993
Diabetes, no. (%)	26 (24.8)	18 (33.3)	8 (15.7)	0.0342
Pulmonary disease, no. (%)	28 (26.7)	15 (27.8)	13 (25.5)	0.7910
Tobacco use, no. (%)	79 (75.2)	42 (77.8)	37 (72.6)	0.5350
Renal disease, no. (%)	17 (16.2)	10 (18.5)	7 (13.7)	0.5040
Dyslipidemia, no. (%)	71 (67.6)	35 (64.8)	36 (70.6)	0.5271
Hostile abdomen, no. (%)	30 (28.6)	12 (22.2)	18 (35.3)	0.1376
ASA class III, no. (%)	55 (52.4)	30 (55.6)	25 (49.0)	0.5026
Antiplatelet agents, no. (%)	34 (32.4)	19 (35.2)	15 (29.4)	0.5271
Anticoagulated, no. (%)	10 (9.5)	3 (5.6)	7 (13.7)	0.1496

**Table 2:** Baseline aneurysm characteristics, including sac diameter, neck diameter, and neck angle, compared between the <60° and ≥60° groups.

	Aorfix (N = 105)	<60° (n = 54)	≥60° (n = 51)	*p*-Value
Sac diameter, mm	51.6 ± 7.1	50.3 ± 5.6	53.0 ± 8.2	0.0560
Proximal neck diameter, mm	20.2 ± 2.7	20.7 ± 2.5	19.6 ± 2.9	0.0521
Proximal neck length, mm	34.5 ± 15.1	33.3 ± 13.9	35.9 ± 16.4	0.3771
Patients with neck length <15 mm	4 (3.8)	3 (5.6)	1 (2.0)	0.3245
Proximal neck angle, °	54.8 ± 29.2	30.7 ± 15.1	80.3 ± 15.6	<0.0001
Patients with neck angle, ≥90°	15 (14.3)	-	15 (29.4)	<0.0001

**Table 3:** Intraoperative outcomes and procedural data, including procedure duration, blood loss, contrast volume, and adjunctive procedures.

	Aorfix (N = 105)	<60° (n = 54)	≥60° (n = 51)	*p*-Value
Primary technical success, no. (%)	103 (100)	50 (100)	54 (100)	
Procedure duration, mean ± SD, min	154.1 ± 51.0	140.1 ± 47.4	168.9 ± 50.9	0.0034
Bleeding, mean ± SD, ml	132.1 ± 168.4	146.0 ± 205.6	117.5 ± 117.2	0.3875
Contrast volume, mean ± SD, ml	177.9 ± 50.5	167.7 ± 47.2	189.5 ± 52.0	0.0301
Fluoroscopy time, mean ± SD, min	46.7 ± 22.1	38.0 ± 17.2	56.1 ± 23.0	<0.0001
IMA occlusion, no. (%)	54 (51.4)	31 (57.4)	23 (45.1)	0.2066
IMA embolization	20 (19.0)	11 (20.4)	9 (17.7)	0.7222
Aortic cuff first	35 (33.3)	20 (37.0)	15 (29.4)	0.4068
IIA embolization, no. (%) Unilateral Bilateral	48 (45.7) 41 (39.0) 7 (6.7)	25 (46.3) 21 (38.9) 4 (7.4)	23 (45.1) 20 (39.2) 3 (5.9)	0.9514
IIA revasculization, no. (%)	17 (16.2)	5 (9.3)	12 (23.5)	0.0449
Necessary adjunctive procedures, no. (%)				
Access site PTA	17 (16.2)	6 (11.1)	11 (21.6)	0.1440
Renal angioplasty	7 (6.7)	5 (9.3)	2 (3.9)	0.2650
Proximal extension cuff	8 (7.6)	2 (3.7)	6 (11.8)	0.1129

SD, standard deviation; IMA, Inferior mesenteric artery; IIA, internal iliac artery; PTA, percutaneous transluminal angioplasty.

**Table 4:** Thirty-day postoperative outcomes, including freedom from major adverse events (SVS MAE), endoleaks, and graft migration.

	Aorfix (N = 105)	<60° (n = 54)	≥60° (n = 51)	*p*-Value
30-day freedom from SVS MAE, %	1 (1.0)	0	1 (2.0)	0.2278
30-day Type 1 EL, no. (%)	1 (1.0)	0	1 (2.0)	0.2278
30-day Type 2 EL, no. (%)	25 (23.8)	12 (22.2)	13 (25.5)	0.4367
30-day endograft migration, no. (%)	0	0	0	-

EL, endoleaks; SVS MAE, society for vascular surgery major adverse events.

**Table 5:** Aneurysm sac morphology changes (shrinkage and expansion) over a 5-year follow-up period.

	Total (N = 105)			<60° (n = 54)			≥60° (n = 51)		
	1 Year	3 Years	5 Years	1 Year	3 Years	5 Years	1 Year	3 Years	5 Years
Sac shrinkage (≥5 mm), %(n)	57.8% (52/90)	57.4% (31/54)	68.7% (11/16)	54.0% (27/50)	62.5% (20/32)	76.9% (10/13)	62.5% (25/40)	50.0% (11/22)	33.3% (1/3)
Sac expansion (≥5 mm), %(n)	0% (0/90)	5.6% (3/54)	6.3% (1/16)	0% (0/50)	3.1% (1/32)	7.7% (1/13)	0% (0/40)	9.1% (2/22)	0% (0/3)
*p*-value	0.4164	0.5147	0.2024						

**Table 6:** Incidence of Types 1, 2, and 3 endoleaks over a 5-year period, stratified by neck angulation group.

	Total (N = 105)			<60° (n = 54)			≥60° (n = 51)		
	1 Year	3 Years	5 Years	1 Year	3 Years	5 Years	1 Year	3 Years	5 Years
Type 1 or 3 EL, %(n)	0 (0/89)	0 (0/53)	0 (0/17)	0 (0/49)	0 (0/31)	0 (0/14)	0 (0/40)	0 (0/22)	0 (0/3)
Type 2 EL, %(n)	26.7% (24/90)	22.2% (12/54)	18.8% (3/16)	26.5% (13/49)	21.9% (7/32)	15.4% (2/13)	26.8% (11/41)	22.7% (5/22)	33.3% (1/3)
*p*-value	0.9745	0.9410	0.4972						

## Data Availability

Data supporting the findings of this study are available from the corresponding author upon reasonable request.

## Abbreviations

The following abbreviations are used in this manuscript:

AAA	abdominal aortic aneurysm
ARBITER-II	Aorfix Bifurcated Safety and Performance Trial: phase II, angulated vessels
IAA	iliac artery aneurysm
CT	Computed Tomography
EIA	external iliac artery
EVAR	endovascular aneurysm repair
IFU	instructions for use
IIA	internal iliac artery
PYTHAGORAS	Prospective Aneurysm Trial: High Angle Aorfix Bifurcated Stent Graft
SMA	superior mesenteric artery
